# Low-energy adsorptive separation by zeolites

**DOI:** 10.1093/nsr/nwac064

**Published:** 2022-04-05

**Authors:** Ruobing Bai, Xiaowei Song, Wenfu Yan, Jihong Yu

**Affiliations:** State Key Laboratory of Inorganic Synthesis and Preparative Chemistry, College of Chemistry, Jilin University, Changchun 130012, China; International Center of Future Science, Jilin University, Changchun 130012, China; State Key Laboratory of Inorganic Synthesis and Preparative Chemistry, College of Chemistry, Jilin University, Changchun 130012, China; State Key Laboratory of Inorganic Synthesis and Preparative Chemistry, College of Chemistry, Jilin University, Changchun 130012, China; State Key Laboratory of Inorganic Synthesis and Preparative Chemistry, College of Chemistry, Jilin University, Changchun 130012, China; International Center of Future Science, Jilin University, Changchun 130012, China

**Keywords:** zeolites, adsorptive separation, olefin/paraffin separation, CO_2_ separation, hydrogen isotope separation

## Abstract

Separation of mixture is always necessarily required in modern industry, especially in fine chemical, petrochemical, coal chemical and pharmaceutical industries. The challenge of the separation process is usually associated with small molecules with very similar physical and chemical properties. Among the separation techniques, the commonly used high-pressure cryogenic distillation process with combination of high pressure and very low temperature is heavily energy-consuming, which accounts for the major production costs as well as 10–15% of the world's energy consumption. To this end, the adsorptive separation process based on zeolite sorbents is a promising lower-energy alternative and the performance is directly determined by the zeolite sorbents. In this review, we surveyed the separation mechanisms based on the steric, equilibrium, kinetic and ‘trapdoor’ effect, and summarized the recent advances in adsorptive separation via zeolites including CO_2_, light olefins, C_8_ aromatics and hydrogen isotopes. Furthermore, we provided the perspectives on the rational design of zeolite sorbents for the absolute separation of mixtures.

## Teaser

This paper reviews recent advances in zeolite-based adsorptive separation of CO_2_, light olefins, C8 aromatics, and hydrogen isotopes in terms of the steric, equilibrium, kinetic, and ‘trapdoor’ effect. The authors also point out the way toward rational design of zeolite sorbents for challenging separations.

## INTRODUCTION

Matter existing in the earth is usually in the state of gas, liquid and solid, and in the form of either pure substance or mixture. In principle, there is no absolute ‘pure substance’ under usual conditions and the so-called ‘pure substance’ often contains impurities below a given limit. In practice, ‘highly pure substance’ is probably a better description for such matter. For some specific occasions such as semiconductor manufacturing and polymers production, highly pure substance is required. To obtain such substance, separation of the components from a mixture is needed, which can be defined as a process that transforms a mixture into single products by utilizing the difference in the properties of components such as boiling point, size, polarity and affinity. Distillation, usually combining high pressure and very low temperature, is a boiling-point-based separation technology, while adsorptive separation is a size-based, polarity-based and affinity-based separation process. Such a separation/purification process is difficult to achieve because it is against the second law of thermodynamics and therefore needs intensive energy input, which accounts for the major production costs in fine chemical, coal chemical, petrochemical and pharmaceutical industries as well as 10–15% of the world's energy consumption [1,2]. In 2016, Sholl and Lively summarized seven chemical separations that could change the world [3]. Among these separations, ‘alkenes from alkanes’, ‘greenhouse gases from dilute emissions’ and ‘benzene derivatives from each other’ as well as the hydrogen isotopes of D_2_ and T_2_ from H_2_ can be achieved via adsorptive separation—a promising lower-energy alternative to the high-pressure cryogenic distillation process currently used.

Light olefins including ethylene, propylene and 1,3-butadiene are key feed stocks for plastics such as polyethylene (PE)/polypropylene (PP) and polymers, and are produced in the scale of several million metric tons each year. Steam cracking of hydrocarbons and methanol to olefins are two main production processes for light olefins. However, both processes do not produce neat light olefins but also yield a lot of other hydrocarbons such as paraffins and alkynes. Polymerization of light olefins to make plastics and polymers requires highly pure (polymer-grade) olefins in which the concentration of impurities of paraffins or alkynes is usually less than several parts per million (ppm). Currently, separation of light olefins such as ethylene and propylene from the corresponding paraffin of ethane and propane relies on the energy-intensive cryogenic distillation under high pressure, which usually requires the large columns containing >100 trays [4], while separation of light olefins from alkynes (acetylene, propyne and butyne) is achieved by the partial hydrogenation of alkynes over supported Pd-catalysts suffering from poor selectivity and high costs [5]. Therefore, developing alternative technologies with enhanced energy efficiency for such separation is highly desired. Compared to the high-energy-consuming cryogenic distillation process, adsorption-based separation is believed to be an energy-efficient and cost-efficient alternative technology. However, designing appropriate adsorbents to separate the light olefins from the corresponding paraffins or alkynes with very small difference in size and very similar physical properties is still highly challenging.

Carbon dioxide (CO_2_) is a well-known greenhouse gas that can reserve the heat of Earth adsorbed in the daylight. A high concentration of CO_2_ in the atmosphere can significantly warm up Earth and lead to global warming, ocean acidification and other environmental concerns [6]. Researches revealed that the trend of the increase of CO_2_ concentration [7] is consistent with that of the global average temperature increase from pre-industrial levels [8], which triggered the global efforts to reduce the concentration of CO_2_ in the atmosphere. Eventually, reducing CO_2_ emissions into the atmosphere depends on CO_2_ capture from CO_2_ sources and the utilization or storage of captured CO_2_, which is usually called carbon capture, utilization and storage. The point sources of CO_2_ emission include: (i) the flue gas mixture of coal-fired power plants, which contains primarily N_2_, CO_2_, water vapor (H_2_O) and O_2_; (ii) key industrial processes such as cement manufacturing, iron and steel production and petrochemical plants; (iii) natural gas, landfill gas and biogas upgrading, which involves the removal of CO_2_ from CH_4_ to increase the gas purity, preventing the corrosion of industrial facilities and achieving the transport specifications or criterion of making liquefied natural gas (LNG); (iv) the Fischer−Tropsch process; and (v) enclosed environments such as spacecraft/submarine cabins or indoor air in commercial buildings. The currently mature technology for the removal of CO_2_ from large-scale sources of CO_2_ is amine scrubbing (aqueous alkanolamine solutions), which is highly selective for CO_2_ but is also heavily energy-consuming in the regeneration process because CO_2_ molecules are chemically adsorbed by amine molecules and an intensive energy input is needed to break the chemical bonds formed between CO_2_ and the amine for regeneration [9,10]. In addition, the amine solution used in this technology is also severely corrosive toward the equipment [11]. The drawbacks of such technology have stimulated the search for alternative physical adsorption-based methods to separate CO_2_ from N_2_ and CH_4_. So far, several physical adsorption-based technologies have been proposed [12] such as pressure-swing adsorption (PSA) [13,14], vacuum-swing adsorption (VSA) [15,16], temperature-swing adsorption (TSA) [17], as well as membrane-based separation techniques [18]. Among the proposed technologies, the PSA technology has the advantage of low energy consumption and low costs because CO_2_ molecules are selectively adsorbed via the physical interaction on an adsorbent such as zeolites at moderate to high pressures and released upon decreasing the pressure. However, PSA has not been commonly used for the large-scale recovery of CO_2_ from flue gas because of the limitations on certain adsorbents and process technology [12].

C_8_ aromatics including ethylbenzene (EB) and three xylene isomers, i.e. *para*-xylene, *ortho*-xylene and *meta*-xylene are the important raw materials and key precursors for the production of many important chemical intermediates and polymers such as polyethylene terephthalate, polyester and polystyrene, which are mainly produced in a mixture by the catalytic reforming of crude oil, gasoline pyrolysis and toluene disproportionation [19,20]. Due to the very similar size and boiling points of C_8_ aromatics, their separation via conventional methods such as distillation is very difficult. Currently, selective adsorption on zeolites via large-scale simulated moving bed units are the main methods used in industry [21]. To further decrease the energy consumption in the adsorptive separation of C_8_ aromatics, the key is to develop adsorbents with higher efficiency and great efforts have been made worldwide [20,22].

Hydrogen (H, ^1^H) has two isotopes, i.e. deuterium (D, ^2^H) and tritium (T, ^3^H). Deuterium has been widely used in industrial and scientific research including in isotopic tracing [23], neutron scattering [24,25], healthcare and medical treatment applications [26] and nuclear fusion [27]. Tritium can be used in the armament industry and in analytical chemistry as well as in nuclear reactors. However, the release of tritium into the environment is a huge threat to human health. On the basis of the increasing global demand for deuterium and concern for the environmental safety of tritium leaking, development of separation technology for hydrogen isotopes is highly needed. Because of the nearly identical physical and chemical properties of hydrogen isotopes, their efficient separation is extremely difficult and has become one of the greatest challenges in modern separation technology. Currently, the methods developed for the separation of hydrogen isotopes include cryogenic distillation [28,29], the Girdler sulfide process [29] and the chromatographic and thermal cycling absorption process (TCAP) [30]. The Girdler sulfide process and H_2_ cryogenic distillation have been applied at the industrial plant scale. However, these techniques are high-energy-intensive and time-intensive with a relative low separation factor. The separation of hydrogen isotopes under moderate conditions with high selectivity and low cost is highly desired but has remained a huge challenge so far. Quantum sieving, first proposed in 1995 by Beenakker *et al.* based on a model of hard spheres in a hard cylindrical well, has attracted great interest for the separation of hydrogen isotopes, in which the quantum effects on the molecular adsorption and diffusion become significant if the difference between the pore size and the molecular hard core is comparable to the de Broglie wavelength [31]. However, such a separation process also requires the cryogenic temperature [32].

Considering that the adsorptive separation/purification process is a promising lower-energy alternative to the high-energy-intensive process currently used in industrial separation and most of the seven chemical separations changing the world can be achieved via adsorptive separation/purification, we briefly review the recent advances in adsorptive separation via zeolite adsorbents alongside historical overviews on the separation mechanism and provide perspectives for future development, especially discussing the characteristics of the ideal zeolite adsorbents for commercial adsorptive separation toward high selectivity, large adsorption capacity, fast adsorption/desorption kinetics, easy regeneration, long stability and durability, and low cost.

## FUNDAMENTALS AND ADVANCES OF ADSORPTIVE SEPARATION IN ZEOLITES

The term ‘zeolite’ was originally coined by Swedish mineralogist Axel Fredrik Crönstedt to name an aluminosilicate mineral that produced large amounts of steam upon rapidly heating [33]. Later, zeolite was used to refer to crystalline microporous aluminosilicates and the framework of zeolites is constructed by corner-sharing TO_4_ (T = Si or Al) tetrahedra, forming periodic and highly stable 1D to 3D channels with a unique porous structure and aperture size of typically <2 nm, covering the size of most industrially important small molecules [34–36]. Today, the type of framework element of T is further extended to P, Fe, B, Ga, Ti, Ge, etc. and 255 zeolite structures have been recognized by the International Zeolite Association-Structure Commission [37].

In the framework of zeolites, each AlO_4_ tetrahedron imparts a negative charge to the framework that is balanced by an extra-framework cation such as H^+^, Na^+^, K^+^, Ca^2+^, Ni^2+^, Co^2+^, Cu^2+^, etc. within the pore space or at the site of the window. Despite the existence of such cations, the rest of the room in the cavity or channel of the zeolite can still accommodate neutral atoms and molecules that are small enough via the pore windows. In the TO_4_ tetrahedra, the O-T-O angle is close to the tetrahedral angle, i.e. 109.4° [38], while the average angle of the T-O-T bond between two tetrahedra is 145° [39]. Thus, the surface of the channel or pore within the framework is essentially O atoms, whereas the T atoms of Si, Al, P, etc. are buried (recessed) in the tetrahedra of O atoms, which are not fully exposed and accessed by the small adsorbate species. The anionic O atoms are much more polarizable than the Al and Si atoms, making the unique surface chemistry of the internal wall of the pores within the framework of zeolites. Besides the anionic O atoms, cations are located at certain sites that are determined by the topology of zeolites and the location of AlO_4_ tetrahedra. Some of these sites may be inaccessible to the adsorbed small molecules, while some other sites located above the oxide surface are fully accessible and can interact with the adsorbed small molecules.

**Scheme 1.**
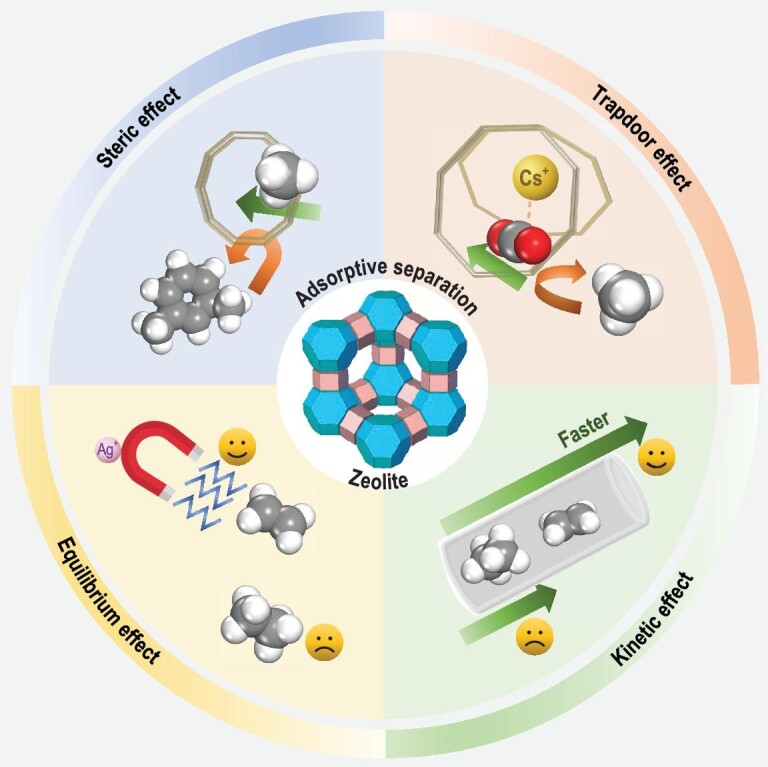
Schematic illustration of the adsorptive separation mechanisms by zeolites.

Imaging an adsorbed small molecule travelling inside the channel or pore inside the zeolite, its diffusion behavior will be affected by factors including: (i) the size, shape and connectivity of the channel or pore, i.e. the topology of the zeolite; (ii) the nature and number of cations of the zeolite framework; and (iii) the type of T atom of the zeolite framework. On the basis of the structural characteristics of zeolites, the adsorptive separation mechanism was originally classified as a steric, kinetic or equilibrium effect [40]. Quantum sieving, discovered in 1995, can be classified into a kinetic effect mechanism. Later, the ‘trapdoor’ or ‘molecular trapdoor’ effect mechanism was proposed upon the investigation of the separation of gases of CO_2_/CH_4_ and CO/N_2_ using chabazite zeolites (**CHA**) [41]. Scheme 1 shows the aforementioned four adsorptive separation mechanisms.

### Steric effect/size exclusion/sieving effect

The steric effect is also referred to as the size-exclusion effect or sieving effect in which only small and properly shaped molecules can be diffused into the zeolite adsorbents, whereas all other molecules and components that are too large are excluded, which can lead to an unlimited high selectivity. Such an effect is unique with zeolites due to the uniform aperture size of pores or channels in the crystalline structure of zeolites. The aperture size (also known as the pore size or window size) is the intrinsic property of a given zeolite because of the robust nature of its open framework. For a given mixture to be separated, the first step is to select an appropriate zeolite in which the maximum aperture size of the pores is between the molecular sizes of the two components. However, the choice is very limited because the aperture size of the pores is mainly controlled by the topology of the open framework of the zeolites. For a zeolite that roughly meets the criterion, precise tuning of the pore or channel structure might be needed to achieve an acceptable selectivity because of the very small difference in the molecular sizes of the aforementioned mixtures. Thus far, several strategies have been developed to precisely tune the pore size of a given zeolite: (i) modifying the accessible room via changing the type of cation or introducing an organic entity [42]; (ii) changing the shape of the pore by relocating the guest species of the cation and/or water molecules [43]; and (iii) shrinking or expanding the aperture size via changing the T–O bond length (i.e. tuning the Si/Al ratio for aluminosilicate zeolites or making pure silica or aluminophosphate zeolites) [44–46].

Zeolite A (LTA) is the classical example in which the pore aperture size can be finely tuned by modifying the type of cations [42]. In 1953, Milton from Union Carbide filed the patent on zeolite A [47] and Breck reported the structure of zeolite A in 1956 [48]. The mutually perpendicular, straight 3D channel system of zeolite A is created by the 11.4-Å cavities (*lta* or α cages) interconnected by six eight-membered ring (8MR) windows with a diameter of 4.1 Å and an effective aperture size of 3.8 Å, which is the origin of the name of 4A for zeolite A with sodium counter cations [37]. When exchanged for Ca^2+^, there are four Ca^2+^ and four Na^+^ in each *lta* cage, which all occupy the center of 6MRs while the center of six 8MRs is vacant. Consequently, the apertures of the resultant Ca–A are completely open and capable of admitting molecules with diameters of ∼4.3 Å, which is the origin of the name of 5A for zeolite A with calcium counter cations. However, when the Na^+^ is exchanged for K^+^, the effective aperture size is reduced to ∼3.0 Å and ∼2.9 Å at 77 K due to the larger K^+^, which is the origin of the name of 3A for zeolite A with potassium counter cations.

**Figure 1.**
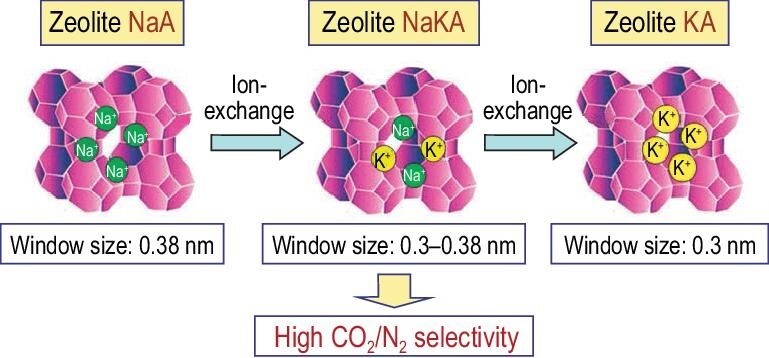
Illustration of the structural mechanism by which K^+^ reduces the effective pore window aperture in NaKA zeolite adapted with permission from [49]. © 2010 The Royal Society of Chemistry.

Complete exchange of Na^+^ ions for K^+^ ions can reduce the aperture size from ∼3.8 to ∼3.0 Å. Thus, partial exchange of Na^+^ ions for K^+^ ions may successively adjust the aperture size of the pore structure. For example, as shown in Fig. 1, Hedin and co-workers systematically adjusted the K^+^ ions content in zeolite A by controlling the exchange degree and successively tuned the aperture size of ∼3.8 Å of 4A to ∼3.0 Å of zeolite 3A [49]. Investigation of the resultant materials on the adsorptive separation of CO_2_/N_2_ indicates that there is an optimal K^+^ content of 17 mol. % in NaKA zeolite for the best performance of selectivity and CO_2_ uptake.

Similarly to the successive tuning of the aperture size of ∼3.8 Å of 4A down to ∼3.0 Å of zeolite 3A via controlling the ratio of Na^+^/K^+^ in zeolite A, Xiao and co-workers precisely tuned the aperture size of pores in zeolite A to between ∼3.8 and 4.2 Å in 0.2-Å increments via sequential and partial ion exchange of Ca^2+^/Ag^+^ [50]. The resultant Ag-Ca-4A showed nearly ideal molecular sieving of C_2_H_4_/C_2_H_6_ (selectivity of ≤17 568 at 298 K and 100 kPa) and superior C_2_H_4_ uptake of 3.7 mmol g^−1^ under ambient conditions. Both pore-size measurement and molecular modeling corroborate that the pore size of Ag-Ca-4A is tuned to be in between the molecular sizes of C_2_H_4_ and C_2_H_6_ (∼0.28 Å difference in kinetic diameter).

Besides the 8MRs in zeolite A, the 12MRs in zeolite mordenite (**MOR**) can also be precisely narrowed. Very recently, Wang and co-workers developed an unusual ‘acid co-hydrolysis route’ that enabled the slow co-condensation of Fe species with Si/Al precursors in the initial gelation stage and finely control of the Fe doping in the resultant zeolite mordenite [51]. With this route, the authors successfully incorporated Fe ions into the **MOR** framework and the tetrahedral Fe species partially occupied the 12MRs of zeolite **MOR**, which precisely narrowed the 12MRs within the kinetic diameters of gas molecules involved in CO_2_ separation (3–4 Å). The resultant Fe-**MOR**s showed record-high volumetric CO_2_ uptakes; excellent size-exclusive molecular sieving of CO_2_ over Ar, N_2_ and CH_4_; stable recyclability; and good moisture-resistance capability.

**Figure 2.**
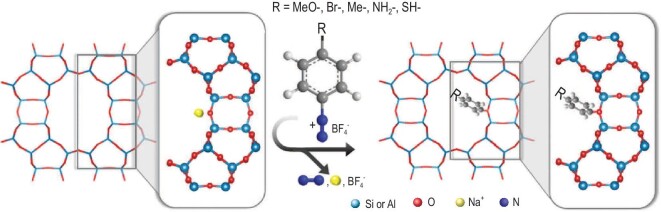
Schematic representation of covalent functionalization of a **MOR** zeolite. Benzene diazonium derivatives (electrophile) are covalently grafted to negatively charged bridging oxygen atoms of micropore walls (nucleophile) via a nucleophilic substitution reaction. N_2_ (blue) and Na^+^BF_4_^−^ are generated as the byproducts. Organoiodide can be used as the grafting agent instead of diazonium derivatives. For clarity, a sodium cation (yellow) is displayed in the structure adapted with permission from [52]. © 2021 Wiley-VCH GmbH.

In addition to the cations inside the channels of zeolites, organic species can also be used to tune the aperture size of pores. Very recently, Jo and co-workers reported a versatile method to finely tune the aperture size of zeolite **MOR**, **LTL**, **FAU** and **MFI** by post-synthesis functionalization with organic molecules of different sizes [52], which is different from the *in situ* organic-functionalization strategy previously reported [53,54]. Figure 2 shows the schematic representation of the covalent functionalization of a **MOR** zeolite. The grafting agents are small and aggressive organic electrophiles of diazonium derivatives and organo-halide, which can access the intracrystalline void space and form the C–O bond via reacting with a bridging oxygen atom of the framework of zeolite. The ideal-adsorbed-solution theory (IAST) selectivity of 4-methoxybenzene-functionalized **MOR** zeolite for the separation of ethylene from ethane was as high as 5873, whereas toluene-grafted **MOR** zeolite can completely separate propylene from propane. At the same time, Liu and co-workers introduced the subunits or fragments of metal-organic frameworks into zeolites and obtained enhanced CH_4_/N_2_ (ZSM-5 and Y) and C_3_H_6_/C_3_H_8_ (**MOR**) separation [55,56]. However, how to avoid the loss of organic functionality during the high-temperature shaping procedure is a big challenge for the practical application of such organic-modified zeolites.

**Figure 3.**
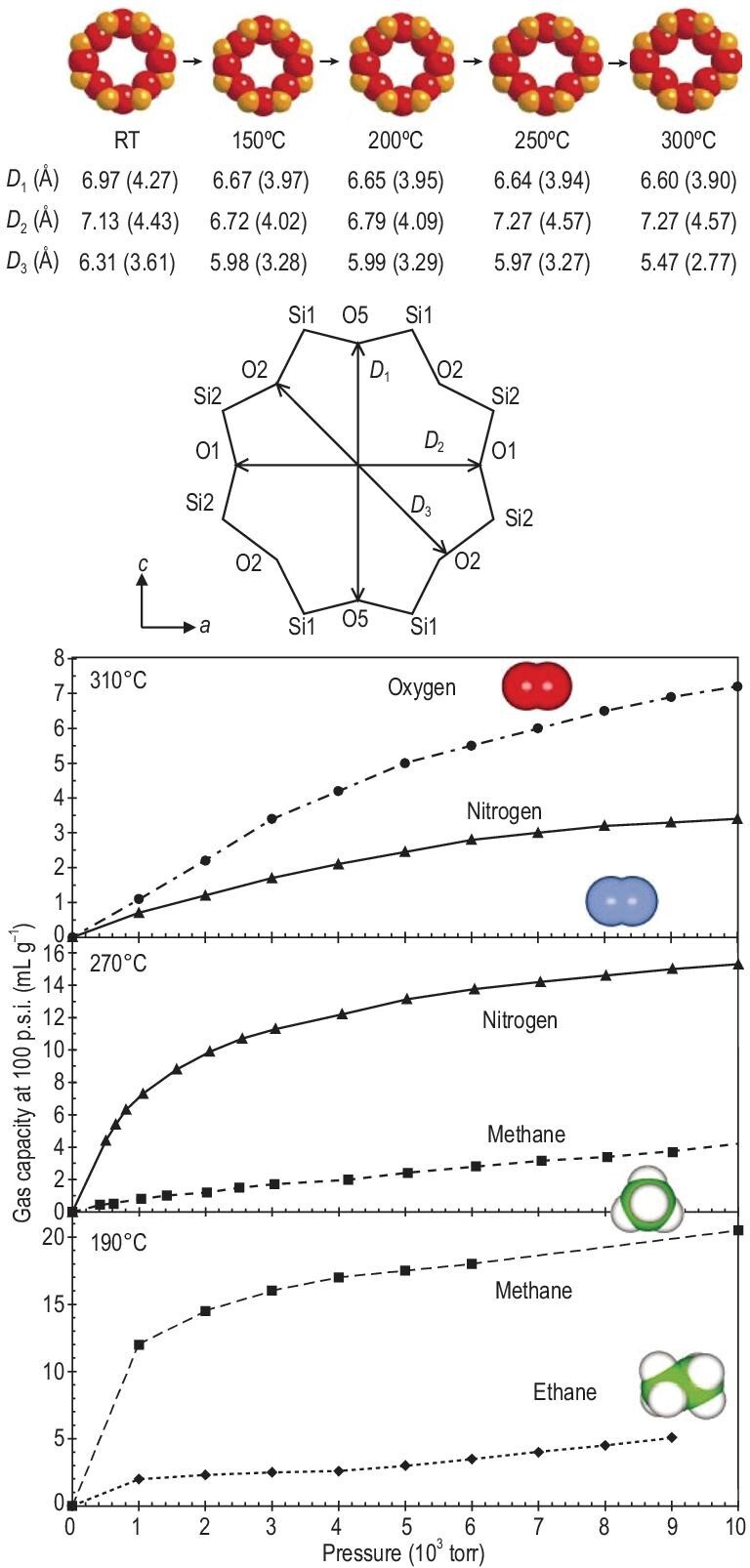
Reduction in the 8MR opening with increasing dehydration temperature and the adsorption isotherms (collected at room temperature) for NaSr-ETS-4 (partially (75%) Sr-exchanged ETS-4) dehydrated at 190°C, 270°C and 310°C demonstrate that the corresponding contracted titanosilicates show selectivity for methane over ethane, nitrogen over methane and oxygen over nitrogen, respectively adapted with permission from [43]. © 2001 Macmillan Magazines Ltd.

Counter cations and water molecules in the channels of zeolites have a strong interaction with the bridging oxygen atom of a framework via Coulomb or hydrogen-bonding interaction. Thus, relocating the counter cations or dehydration may change the shape of pores or channels and further modify the aperture size. The classical example having such phenomenon is the first member of microporous titanium silicate ETS-4 [43], in which the flexible framework can be systematically contracted via dehydration to tune the aperture size of pores. As shown in Fig. 3, the results of the Rietveld refinement of powder neutron-diffraction data revealed a progressive and pronounced shape changing of the 8MR opening in dehydrated samples, in addition to the cation relocation. During the dehydration at elevated temperature, cation relocation took place and two new cation positions appeared. Both shape changing (i.e. pore narrowing) of the 8MR and cation relocation led to the progressive reduction of the aperture size of 8MR. As a result, along with the dehydration temperature being increased from 190°C to 270°C and further to 300°C, ethane (C_2_H_6_), methane (CH_4_) and nitrogen (N_2_) with progressively smaller dimensions were sequentially excluded from adsorption into the crystals (Fig. 3). On the basis of the adsorption and structural results, the authors claimed that the contraction of 8MRs and concomitant cation relocation were primarily responsibility for the gradual exclusion of smaller and smaller molecules. Recently, Wright and co-workers observed that the unit cell volumes decreased by 9.8%, 7.7% and 7.1% for Na-**MER**, K-**MER** and Cs-**MER** (Merlinoite) zeolites with Si/Al = 3.8 upon dehydration [57]. The resultant dry K-**MER** zeolite showed good uptake of CO_2_ at 1 bar and 298 K, rapid adsorption and desorption kinetics, and promising CO_2_/CH_4_ separation.

In addition to the aforementioned strategy to tune the aperture size of zeolites, changing the bond length of the T–O bonds in the framework may also modify the effective size of pores. Zeolite A is a common adsorbent for the adsorptive separation. The Si/Al ratio of zeolite A is usually 1 when crystallized from the organic-free synthetic system and is >1 when crystallized in the presence of organic structure-directing agents. Besides the aluminosilicate form, pure silica form (ITQ-29) and aluminophosphate form (AlPO-**LTA**) zeolite A were also discovered with the assistance of supramolecular self-assembled molecules and diaza-polyoxa-macrocycle as organic structure-directing agents, respectively [58,59]. Molecular mass transfer studies revealed that the diffusivities of small molecules in ITQ-29 and AlPO-**LTA** were significantly smaller than in the zeolite NaCaA [45], due to the slight reduction in the window apertures of SiO_2_ and AlPO_4_ forms [60].

For a given separation process, available zeolites with appropriate aperture size are very limited, especially for small-pore zeolites (i.e. with 8MR windows). Even for an appropriate zeolite, there is usually a trade-off between the selectivity and adsorption capacity. To overcome this problem, making core–shell zeolite composites might be a feasible strategy, with the adsorbent with high selectivity as the shell and high adsorption capacity as the core. For example, Laroche and co-workers prepared a zeolite beta-silicalite-1 composite by growing silicalite-1 shells on single micron-sized zeolite beta crystals (core) [61]. The obtained composite allowed more selective separation of mono-branched and di-branched hexane isomers than either of its components alone and had a significantly higher adsorption capacity than silicalite-1 (shell). Recently, Miyamoto *et al.* grew a silicalite-1 coating on ZSM-5 zeolite crystals to improve the catalytic *p*-xylene selectivity [62]. It was found that the silicalite-1 coating improved the separation factors of *p*-xylene over *o*-xylene and *m*-xylene on silicalite-1/ZSM-5 than on parent ZSM-5.

### Equilibrium effect

Equilibrium separation is based on the interaction between the adsorbate and the adsorbent. The molecules of the adsorbate (targeted molecules) mainly interact with the adsorption sites. When the mixture is adsorbed into the adsorbent, molecules having stronger interaction with sorbent will be preferentially adsorbed, which generates the selectivity of preferentially adsorbed molecules over less preferentially adsorbed molecules. Such preferential adsorption of one component over all other components changes the composition of the mixture and sequentially facilitates the separation of the mixture. For purification, particularly ultra-purification, strong interaction between adsorbate and adsorption sites is needed, which leads to ultra-high product purity. However, strong interaction always indicates a high regeneration energy. For aluminosilicate zeolites, counter cations are uniformly dispersed above the negatively charged oxides on their surfaces. Cations with high valences (i.e. charges) and small ionic radii (i.e. high charge density) would result in strong interactions with the adsorbate.

Zeolite NaX (or 13X) has been used commercially for air separation to make pure oxygen via PSA since the 1970s. Li^+^ ions exchanged low silica zeolite X with a Si/Al = 1 (denoted as Li-LSX) is the best adsorbent that is commercially available today [63]. Cation sites in LSX are distributed in the center of the double six-membered ring (D6R), above the single six-membered ring (S6R) in supercage and cuboctahedron, and near the four-membered ring (4MR) in supercage. Only the sites exposed to the supercage cavity can have the interaction with the adsorbate [40]. Upon the complete ion exchange of Na^+^ with Li^+^, the resultant Li-LSX preferentially adsorbs N_2_ over O_2_ and thus significantly improves N_2_/O_2_ selectivity because the ionic radius of Li^+^ (0.68 Å) is smaller than that of Na^+^ (0.97 Å) and subsequently interacts much more strongly with N_2_ [63]. Taking such advantage, O_2_ with 95+% can be produced from air by a PSA procedure.

Besides the physical interaction (i.e. non-bonding interaction), weak chemical interaction such as π-complexation that is stronger than those physical interactions can also exist between the specific targeted molecules and the adsorption site, and thus may be used to enhance the separation selectivity and adsorption capacity. Unlike the strong chemical interaction of covalent bonds, the weak chemical interaction of π-complexation is still weak enough to be broken by reduce the pressure or an increase in the temperature as operated in the PSA or TSA procedure [40]. To create the π-complexation interaction between the adsorbent and the adsorbate, the adsorption sites of the adsorbent should have the ability to form the usual σ bonds with their s-orbitals to the adsorbate; in addition, their d-orbitals can back-donate electron density to the anti-bonding π-orbitals of the adsorbate, which pertain to the main group (or d-block) transition metals. In tailoring adsorbents for π-complexation, the bond strength between the adsorbate and the adsorption sites (cations) as well as the total number of cations (i.e. the density of cations) are two important parameters. The latter depends on the cation exchange capacity of the zeolite (i.e. Si/Al ratio for aluminosilicate zeolites and Si content for silicoaluminophosphate (SAPO) zeolites).

On the basis this strategy, Li and co-workers introduced the isolated open nickel(II) in the 6MR of zeolite Y (**FAU**) as the adsorption site to highly selectively remove alkyne impurities for the production of polymer-grade light olefins [64]. Under ambient conditions, the as-prepared Ni@**FAU** composite showed preferential adsorption of alkynes and efficient separations of acetylene/ethylene, propyne/propylene and butyne/1,3-butadiene mixtures, with unprecedented dynamic separation selectivities of 100, 92 and 83, respectively. *In situ* neutron-diffraction and inelastic neutron-scattering results revealed that nickel(II) formed the metastable [Ni(II)(C_2_H_2_)_3_] complexes with acetylene. Later, the same group prepared atomically dispersed copper(II) sites in zeolite Y and achieved the efficient separation of acetylene and CO_2_ under ambient conditions, which is an extreme challenge in industry due to the almost identical molecular sizes and volatilities of acetylene and CO_2_ [65]. High-purity acetylene (98–99%) from the mixture of acetylene and CO_2_ was obtained and the separation factor was 22.2 with a dynamic uptake of acetylene of 1.51 mmol g ^−1^ at 298 K. Characterizations of *in situ* neutron powder diffraction and inelastic neutron scattering revealed that there was chemoselective yet reversible binding between the copper(II) site and acetylene, whereas adsorbed CO_2_ was stabilized by weak host–guest interactions with the framework oxygen, thus resulting in the efficient separation of these two gases under flow conditions.

**Figure 4.**
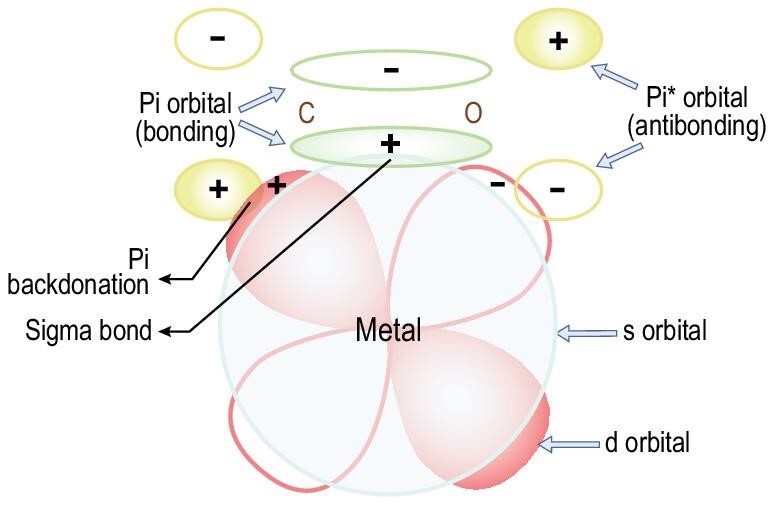
Pi-complexation interaction between a CO_2_ and a transition-metal ion by forming sigma bond (electrons on the Pi orbital of CO_2_ donated to the s orbital of the transition metal) and Pi back-donation (electrons on the d orbital of the transition metal back-donated to the Pi^*^ orbital of the CO_2_) adapted with permission from [66]. © 2019 Elsevier BV.

π-Complexation can be formed not only between the transition cations and the double C=C bonds or triple C≡C bonds in alkyne but also between the transition cations and the CO_2_. Recently, Shang and co-workers introduced the transition metals of Co(II), Ni(II), Zn(II), Fe(III), Cu(II), Ag(I), La(III) and Ce(III) into zeolite SSZ-13 via ion exchange and investigated their CO_2_/N_2_ separation performance by unary static isothermal adsorption and binary dynamic column breakthrough experiments as well as predicted performance in the PSA/VSA procedure [66]. Among these adsorbents, Co(II)/SSZ-13 and Ni(II)/SSZ-13 showed the highest CO_2_ uptake (4.49 and 4.45 mmol g^−1^, respectively) and selectivity of CO_2_ over N_2_ (52.55 and 42.61, respectively) at 273 K and 1 atm. Such outstanding separation performance was attributed to the Pi back-donation exclusively formed between CO_2_ and transition-metal cation sites, as shown in Fig. 4.

Isomorphous heteroatom substitution of the T atom of the framework of zeolites can also alter the property of the framework and tune the interaction between the adsorbate and the adsorbent. For example, Yu, Song and co-workers hydrothermally synthesized Mg, Co-substituted aluminophosphate zeolites with **ERI** framework topology (denoted as MgAPO-**ERI** and CoAPO-**ERI**) by using *N*,*N*,*N^′^*,*N^′^*-tetramethyl-1,6-hexanediamine as the organic template and investigated their CO_2_ adsorption properties in comparison to those of the pure aluminophosphate counterpart AlPO-**ERI** [67]. Their results clearly showed that the incorporation of Mg^2+^ and Co^2+^ ions in the framework of the AlPO-**ERI** greatly improved the adsorption selectivities of CO_2_ over CH_4_ and N_2_. In addition to improve the selectivity, isomorphous heteroatom substitution of Cu^2+^ in microporous silicate conducted by Yoon and co-workers greatly improved the CO_2_ adsorption from the humid flue gases [68]. The H_2_O-specific and CO_2_-specific adsorption sites were created but did not have H_2_O/CO_2_-sharing sites in the highly stable microporous coppersilicate SGU-29. Both H_2_O and CO_2_ were adsorbed from the humid flue gases and atmosphere but the adsorption of H_2_O did not interfere with the adsorption of CO_2_.

Tuning the interaction between the adsorbate and adsorbent is mainly focused on the metal cations and the investigation of the proton (also acid site, Brønsted acid site) in zeolites is rare. Very recently, Van Speybroeck and co-workers studied the influence of acid sites on the diffusion of alkenes through small-pore SAPO-34 zeolites by combining the experimental and theoretical approaches [69]. The results clearly showed that Brønsted acid sites had a promotional effect on the diffusion rate of ethene and propene, whereas the transport of alkanes was insensitive to the acid sites. The enhanced diffusivity of unsaturated hydrocarbons was attributed to the formation of favorable π–H interactions with acid protons, which was confirmed by infrared (IR) spectroscopy measurements. These results demonstrated that the acid site distribution is an important design parameter for optimizing product distributions and separations. At the same time, Hong and co-workers investigated the propylene/propane separation on the dealuminated Cs-ZK-5 zeolite [70], which showed high propylene uptake and propylene/propane selectivity (1.9 mmol g^–1^ and 2.4, respectively) at 298 K and atmospheric pressure, together with fast adsorption kinetics. More interestingly, it was found that no significant loss in uptake or selectivity was observed even after repeated adsorption–desorption cycling under the VSA mode and dynamic breakthrough experiments, which was attributed to the lack of strong acid sites to induce propylene oligomerization at room temperature. Such impressive regeneration is comparable with the behavior of the pure silica small-pore zeolite ITQ-12 [71] and much better than that of Na-A and Ca-A, suggesting the dealuminated Cs-ZK-5 zeolite has great potential in industrial-scale propylene/propane separation.

Tuning the interaction between adsorbate and adsorbent is also successful in hydrogen isotope separation. Recently, Xiong *et al.* reported highly effective hydrogen isotope separation through the dihydrogen bond on Cu(I)-exchanged zeolites from liquid-nitrogen temperature to near room temperature [72]. The Cu^2+^ was introduced into the hydrogen form ZSM-5 (H-ZSM-5) with a Si/Al = 11.5 via a standard ion-exchange procedure. During the activation procedure, Cu(II) was reduced gradually to Cu(I) by a ‘self-reduction’ process as confirmed by the X-ray photoelectron spectroscopy. The resultant Cu(I)-ZSM-5 zeolite has a D_2_/H_2_ selectivity of 24.9—the highest selectivity value ever measured at 100 K. Through only three adsorption/desorption cycles, 99.6% of deuterium can be enriched from a mixture with a deuterium concentration of 2.5%. Such excellent performance was attributed to the strong chemical affinity through the dihydrogen bond on Cu(I) sites with a large isotope effect in zero-point energy (Δ*E*_ZPE_) and adsorption enthalpy (Δ*H*).

### Kinetic effect

Kinetic separation is achieved by virtue of the differences in the diffusion rates of different molecules, which is also known as partial molecular sieve action and treated as an alternative when equilibrium separation is not feasible. In some cases, the amounts of different components of a mixture adsorbed at equilibrium are similar, although some components may diffuse faster than others. Such different diffusing rates can be used to separate the components. For kinetic separation, the pore size of the adsorbent needs to be precisely tailored to lie between the kinetic diameters of the two molecules that are to be separated [40].

The remarkable example of kinetic separation by zeolites is the separation of ethylene and ethane by a flexible pure silica zeolite (ITQ-55) reported by Corma and co-workers [73]. ITQ-55 was synthesized either in alkaline conditions (OH^–^ media) or using F^–^ anions as mobilizer agents of the silica (F^–^ media) with a bulky organic structure-directing agent of *N*^2^,*N*^2^,*N*^2^,*N*^5^,*N*^5^,*N*^5^,3a6a-octamethyloctahydropentalene-2,5-diammonium dihydroxide. The most notable characteristic of ITQ-55 is the presence of twined heart-shaped cages that are interconnected by sharing a common 8MR (5.3 × 3.4 Å). These cavities are accessible through two parallel systems of zigzag 8MR channels (5.9 × 2.1 Å), the only gate for guest molecules to access ITQ-55, which makes ITQ-55 a tortuous monodirectional small-pore system with relatively large cavities. In principle, the distance of 2.1 Å of the short axis of zigzag 8MR makes it impossible for ITQ-55 to adsorb any gas molecule. However, adsorption data of ITQ-55 on ethylene and ethane showed that adsorption equilibrium for ethylene and ethane on ITQ-55 synthesized in F^–^ media (large crystals) and for ethylene on ITQ-55 synthesized in OH^–^ media (small crystals) was reached, but not for ethane adsorption on ITQ-55 synthesized in OH^–^ media (small crystals). Such surprising results indicate that the framework of ITQ-55 is flexible and the preferential diffusion of ethylene over ethane on ITQ-55 small crystals can be used for the kinetic separation of ethylene/ethane. The distributions of minimal window size were calculated by *ab initio* molecular dynamics simulations to gain insight into the degree of flexibility of the structure of ITQ-55. Simulation results indicate that the mean distance of the short axis of 8MR is 2.38 Å with a standard deviation of 0.17 Å for the empty structure, while such distance expanded to 3.08 Å with a standard deviation of 0.16 Å when a C_2_H_4_ molecule was constrained to the center of the same 8MR. Such results suggest in general that diffusion of tightly fitting molecules in small-pore zeolites is strongly influenced by framework flexibility and the framework flexibility generally accelerates the diffusion of tightly fitting molecules. For ITQ-55, upon admitting the C_2_H_4_ molecule, the gate of 8MR expands ∼1 Å larger than the nominal crystallographic pore aperture size. Multicomponent gas breakthrough experiments confirmed that ITQ-55 can kinetically separate ethylene from ethane with an unprecedented selectivity of ∼100 and the adsorption properties remained unaltered upon exposure to olefins for as long as 3 months. Such stability can be attributed to the lack of acidity in ITQ-55.

Similar to ITQ-55, ITQ-69 is a small-pore germanium-containing zeolite with a 3D small-pore channel system (8 × 8 × 8 MR) reported by Valencia and co-workers very recently [74]. ITQ-69 was synthesized in the form of pure germania as well as silica-germania zeolites with different Si/Ge ratios and was stable upon calcination to remove organic structure-directing agents. The adsorption results clearly showed that propylene diffused much faster than propane through ITQ-69. The ratio of the Fickian diffusion coefficient for propylene and propane is of an order of 10^4^, suggesting that propylene and propane can be kinetically separated using ITQ-69 as an excellent selective adsorbent.

It is usually believed that the crystal size of a zeolite has less influence on the separation performance. However, recent work by Yang *et al*. indicated that the ZK-5 zeolite with reduced crystal size can remarkably enhance CH_4_ adsorption and CH_4_/N_2_ separation performance [75]. The authors reduced the crystal size of ZK-5 zeolite from micron-size (3 μm) to nano-size (50–100 nm) via the regulation effects of β-cyclodextrin. Nano-sized ZK-5 exhibits a superior surface area (370 cm^2^g^−1^) and pore volume (0.22 cm^3^g^−1^) than the micron-sized ZK-5 (149 cm^2^g^−1^ and 0.07 cm^3^g^−1^), which is attributed to the detection of more accessible micropores due to the reduction in crystal size. Compared to the micro-sized ZK-5 and the commercial zeolites, nano-sized ZK-5 has a record-high CH_4_ adsorption capacity (1.34 mmol g^−1^ at 298 K and 1 bar) by 64%. The equilibrium adsorption selectivity of CH_4_/N_2_ (20/80, v/v) on nano-sized ZK-5 is as high as 4.2 (IAST method) at 1 bar and 298 K. The adsorption kinetics experiments illustrated the boosted gas diffusion and mass transfer rate, and the breakthrough experiments confirmed the practical feasibility of the CH_4_ enrichment from low-quality coal-bed gases (CH_4_/N_2_).

Besides the aforementioned examples, Yang *et al.* discovered that the diffusion rate of N_2_ is much faster than that of CH_4_ in Na- and Ca-clinoptilolites (an abundant natural zeolite with a 2D channel structure formed by 8-membered rings and 10-membered rings), leading to an excellent separation performance of N_2_/CH_4_ [76–78]. Later in the early 2000s, Yang *et al.* continuously studied the kinetic separation performance of clinoptilolites with H^+^, Li^+^, Na^+^, K^+^, Mg^2+^, Ca^2+^, Sr^2+^, Ce^3+^ and mixed ion-exchanged clinoptilolites for N_2_/CH_4_ and found that in clinoptilolites with Li^+^, Mg^2+^, Ca^2+^ and Ce^3+^, N_2_ diffuses much faster than CH_4_. Besides, a mixed ion-exchanged clinoptilolite like Mg/Na (50/50) also shows excellent kinetic selectivity for N_2_/CH_4_ separation. Therefore, clinoptilolites are potential adsorbents suitable for N_2_/CH_4_ separation with a PSA process [79,80].

### Trapdoor effect

**Figure 5.**
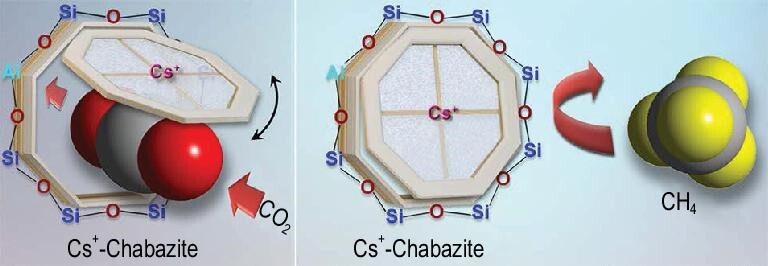
Schematic illustration of the ‘trapdoor’ effect. The CO_2_ molecule is capable of opening the cesium trapdoor to enter the cha cage whereas CH_4_ is excluded. After intrusion of a CO_2_ molecule through the 8MR, the ‘gate’ immediately recloses adapted with permission from [41]. © 2012 American Chemical Society.

The trapdoor effect of zeolites was first observed in a small-pore chabazite (**CHA**) zeolite that can even perform ‘size-inverse’ separation [41]. The framework of a **CHA** zeolite contains large ellipsoidal cavities (*cha* cages) with approximate apertures of 6.7 × 10 Å, which are accessible through 8MRs (3.8 × 3.8 Å), the largest opening in **CHA** [37]. For **CHA** structures with a low Si/Al ratio (<3), K^+^, Rb^+^ or Cs^+^ ions fully occupy the 8MRs and thus affect the accessibility of the *cha* cages. Larger CO molecules have a stronger interaction with the cations than smaller N_2_ molecules, which induces temporary and reversible cation deviation from the center of 8MRs and allows exclusive admission of CO (3.76 Å) instead of N_2_ (3.64 Å). Such separation also gives a high selectivity of 93 for CO_2_/CH_4_ separation over a large pressure range (Fig. 5). The trapdoor-effect-based separation is guest-selective and size-inverse that could help with carbon capture and hydrogen purification.

As aforementioned, the origin of the ‘trapdoor’ effect is from the occupancy of the entrance (usually 8MR) by cations and gas molecules have to move them away from the center of the entrance. Notice that thermal motion of cations always exists above absolute zero and the kinetic energy of thermal motion is directly proportional to the absolute temperature. Thus, above a certain threshold admission temperature, the cation's increased thermal energy allows it to be displaced temporarily by gas molecules with weaker interaction energies, i.e. the ‘trapdoor’ effect is weakened at high temperatures. Considering that the adsorption step of a PSA cycle is strongly exothermic (adiabatic operation) and the flue gas is hot (≤363 K) in real post-combustion carbon-capture processes, there is a strong need to enhance the ‘trapdoor’ effect in the zeolite that can exclusively adsorb CO_2_ at industrial carbon-capture operating temperatures, which needs to enhance the interaction between the cations and the entrance in order to enhance the ‘trapdoor’ effect.

With this knowledge, Du *et al*. synthesized a **CHA** zeolite with increased energy barriers Δ*E* required for the passage of gas molecules through the cation-blocked 8MR entrance [81]. By performing the density functional theory calculations, the authors realized that Δ*E* for a given gas–zeolite system strongly depends on the density of the cations in the trapdoor zeolite. A higher density of cations in the *cha* cage substantially increases the space hindrance and repulsion for the movement of the door-keeping cation as well as the gas molecule. Moreover, the high negative charge density on the aluminosilicate framework can make the cations less mobile. Thus, the authors speculated that reducing the Si/Al ratio in **CHA** zeolite would increase the threshold admission temperature. After optimizing the Si/Al ratio, the authors synthesized a **CHA** zeolite with an optimal Si/Al ratio of 1.9 from fly ash, which can exclusively adsorb CO_2_ at industrial carbon-capture operating temperatures.

**Figure 6.**
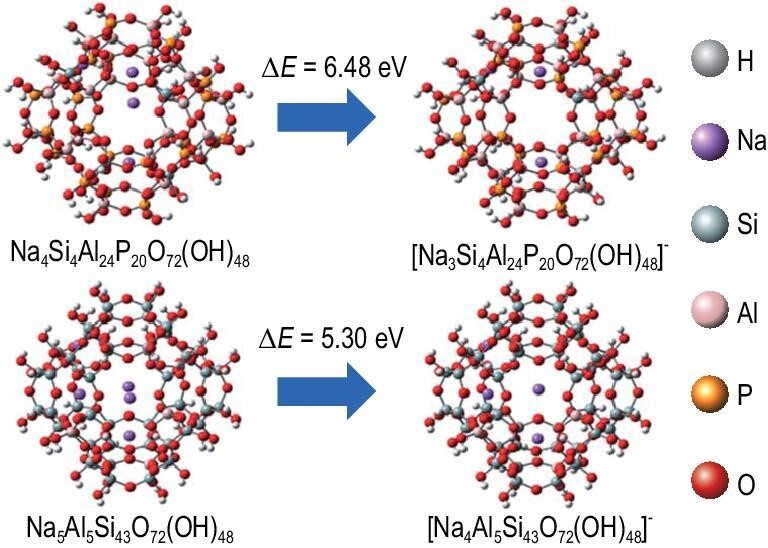
Molecular structures of two neutral Na_5_Al_5_Si_43_O_72_(OH)_48_ and Na_4_Si_4_Al_24_P_20_O_72_(OH)_48_ clusters and the binding energy of Na^+^ in the center of elliptical *s*8*r*s in two clusters adapted with permission from [82]. © 2021 The Royal Society of Chemistry.

Very recently, Yu, Yan and co-workers reported that the Na^+^ form of silicoaluminophosphate zeolite with **RHO** structure (denoted as Na-SAPO-**RHO**) has a stronger ‘trapdoor’ effect than the aluminosilicate counterpart **RHO** zeolite [82]. The SAPO zeolite was prepared using an organic structure-directing agent followed by direct ion exchange giving Na-, K- and Cs-form SAPO-**RHO** zeolites. Adsorption experiments revealed that Na-SAPO-**RHO** exhibited unprecedented separation for CO_2_/CH_4_, superior to all of the nanoporous materials reported to date, including the aluminosilicate counterpart **RHO** zeolite [83]. Rietveld refinement revealed that Na^+^ is sited in the center of the single eight-membered ring (S8R) connecting to the *lta* cages. As shown in Fig. 6, theoretical calculations showed that the interaction between Na^+^ and the S8R in SAPO-**RHO** (Δ*E *= 6.48 eV) was stronger than that in aluminosilicate **RHO** (Δ*E *= 5.30 eV), giving an enhanced ‘trapdoor’ effect and record-high selectivity for CO_2_ with the separation factor of 2196 for CO_2_/CH_4_ (0.02/0.98 bar). Even though the stronger interaction reduces the uptake of CO_2_, the uptake of CH_4_ is significantly hindered by approximately two orders of magnitude, which results in a better selectivity.

‘Trapdoor’ effect as well as the threshold admission temperature have also been utilized in the low-energy hydrogen–deuterium isotope breakthrough separation [84]. Ting and co-workers reported that hydrogen–deuterium isotope separation was achieved using breakthrough separation with a single pass through a packed bed of Cs-chabazite at moderate temperatures (293 K) and pressures (0.17 MPa) when one component was in a sufficiently low concentration. The open or close of the ‘trapdoor’ was controlled by temperature depending on the threshold admission temperature of H_2_ (333 K), above which the trapdoor is considered ‘open’ and below which it is considered ‘closed’. Such a ‘trapdoor’ effect-based separation process would significantly lower the total energy demand compared with current hydrogen isotope cryogenic distillation separation techniques and can be applied to the separation of low concentrations of D_2_ (0.0156%) present in standard-grade H_2_.

**Figure 7.**
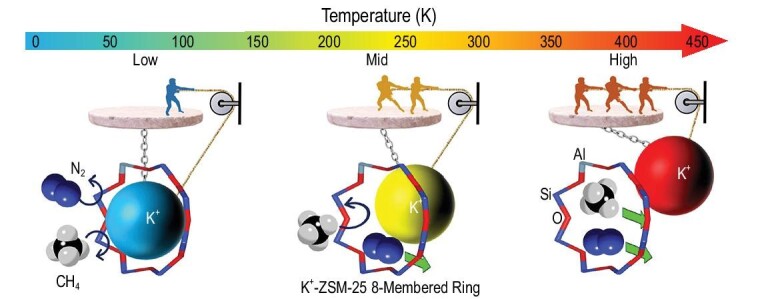
Schematic illustration of the temperature-regulated adsorption of the K-ZSM-25 trapdoor zeolite for removing N_2_ from CH_4_ adapted with permission from [85]. © 2021 American Chemical Society.

Very recently, the threshold admission temperature of the ‘trapdoor’ for N_2_ and CH_4_ molecules was used by Zhao *et al*. to remove N_2_ from CH_4_ through K-ZSM-25 zeolite, as shown in Fig. 7 [85]. The outstanding N_2_/CH_4_ selectivity is achieved within a specific temperature range (240−300 K) where the thermal motion of K^+^ provides enough space only for the relatively smaller molecule (N_2_) to diffuse into and through the zeolite supercages while the relatively larger molecule (CH_4_) was rejected. Such temperature-regulated adsorption of K-ZSM-25 trapdoor zeolite opens up a new approach for removing N_2_ from CH_4_ in the natural gas industry without deploying energy-intensive cryogenic distillation around 100 K.

**Table 1.** Zeolite materials in several adsorptive separation processes.

Process	Mechanism	Materials	Performance	References
Olefin/paraffin separation	Steric effect	Ag-Ca-4A	C_2_H_4_/C_2_H_6_ selectivity of 17 568 at 298 K and 100 kPa	[50]
		Zn**MOR**-pIM	C_3_H_6_/C_3_H_8_ selectivity of 139 at 298 K and 50 kPa	[56]
		MeOPh-f-MOR	IAST C_2_H_4_/C_2_H_6_ selectivity of ≈5873	[52]
		Dealuminated Cs-ZK-5	C_3_H_6_/C_3_H_8_ selectivity of 2.4 at 298 K and 1 atm	[70]
	Kinetic effect	ITQ-55	C_2_H_4_/C_2_H_6_ selectivity of 50 at 112°C	[73]
CO_2_ separation	Steric effect	NaKA	CO_2_/N_2_ selectivity of 172 at 298.15 K and 0.85 bar (17 at.% K^+^ content)	[49]
		Fe-**MOR**	CO_2_/CH_4_, CO_2_/N_2_ and CO_2_/Ar selectivities of 298.9, 51.8 and 23.8 at 298 K and 1 bar	[51]
		K-**MER**	Estimated CO_2_/CH_4_ selectivity of 850 at 298 K and 1 bar	[57]
	Equilibrium effect	Cu@**FAU**	C_2_H_2_/CO_2_ separation factor of 22.2 at 298 K	[65]
		Co(II)/SSZ-13	CO_2_/N_2_ selectivity of 52.55 at 273 K and 1 atm	[66]
		MgAPO-**ERI**	CO_2_/N_2_ (0.15:0.85) IAST selectivity of 29.41 at 273 K and 1 bar	[67]
	Trapdoor effect	Cs-**CHA**	CO_2_/CH_4_ selectivity of 109 at 293K and 116 kPa	[41]
		K-**CHA** (Si/Al = 1.9)	CO_2_/CH_4_ and CO_2_/N_2_ selectivities of 583 and 90 at 303 K and 1 bar (50:50)	[81]
		Na-SAPO-**RHO**	CO_2_/CH_4_ and CO_2_/N_2_ selectivities of 2196 and 196 at 298 K	[82]
C8 aromatics separation	Steric effect	Silicalite-1 coated ZSM-5	Low-coverage separation factors of p-xylene/m-xylene and p-xylene/o-xylene at 553 K: 16.7 and 22.7	[62]
Hydrogen isotope separation	Equilibrium effect (chemical affinity quantum sieving)	Cu(I)-ZSM-5	D_2_/H_2_ selectivity of 24.9 at 100 K	[72]
	Trapdoor effect	Cs-chabazite	Breakthrough separation of ^1^H and ^2^H occurs at ∼293 K	[84]

## CONCLUSION AND OUTLOOK

Making products is the responsibility of industry, which needs highly pure substances. Very unfortunately, most matter exists in the form of a mixture. Thus, the separation of matter on a large scale such as light hydrocarbons, C_8_ aromatics and hydrogen isotopes is needed by industry. In addition, large-scale CO_2_ and volatile organic compounds capture is also urgently needed for environmental protection. Most of the current separation techniques for these needs are high-energy-intensive processes and the new separation procedures with low energy consumption are highly desired.

It is well accepted that the adsorptive separation process is a promising lower-energy alternative to the high-energy-intensive process currently used. To this end, the key is adsorbent, which should possess the following characteristics to be commercialized, namely high selectivity, large adsorption capacity, fast adsorption/desorption kinetics, easy regeneration, long stability and durability, and low cost. Among these characteristics, the most important one is selectivity, but there is a trade-off between selectivity and regeneration energy. Combining all these characteristics, zeolites are the most promising candidate. Table 1 summarizes different zeolite materials utilized in several adsorptive separation processes.

To date, there are four adsorptive separation mechanisms for zeolite-based separation processes in terms of the steric, equilibrium, kinetic and ‘trapdoor’ effect. For given separation needs, the first thing to do is to analyse the physical and chemical properties of the target molecules to determine the possible separation mechanism. The first choice is the steric or sieving effect because such a mechanism can reach an unlimited high selectivity. However, finding an available zeolite with the aperture size just between those of two molecules is usually extremely difficult in the existing or modified zeolites. So far, only >250 types of zeolitic structures have been collected in the database by the International Zeolite Association-Structure Committee. Considering that the rational design and synthesis of specific zeolite with desired structure and composition are still huge challenges, selecting the rest of the separation mechanisms is highly possible, i.e. precisely modifying the composition of candidate zeolites with the aforementioned strategies (i.e. modifying the nature and number of cations).

After selecting the zeolite adsorbent, the adsorptive separation studies in the laboratory should be conducted under conditions that are as close as possible to industrial conditions, such as the composition of the mixture, operating temperature and pressure, and humidity level. At the same time, shaping the procedure must be seriously considered because the powder form of an adsorbent cannot be applied in industry. Besides the traditional shaping procedure, new shaping techniques such as 3D printing should be especially paid attention to [86–88]. Once the selectivity meets the minimum requirement, all other characteristics for an adsorbent to be commercialized should be investigated. After combining all results, the next step is to optimize the separation procedure on the basis of economics (e.g. working capacity and selectivity) and sustainability, using a synergistic separation strategy if necessary.

If the laboratory results are good enough, the next step would be scale up for both adsorbent making and procedure testing, which needs the close collaboration of academia and industry engineers. Once it is successful at the pilot scale, the next challenge is application on an industrial scale, which is also highly risky.

Achieving carbon neutrality by the coming middle century is crucial for preventing the global average temperature increase of >1.5 or 2ºC. To that end, replacing the high-energy-consuming cryogenic distillation process with a low-energy adsorptive separation process is critical for such a goal. As the most promising adsorbents among the porous materials, zeolites will have great opportunities and a bright future in the aforementioned extremely challenging separations.

## References

[1] Oak Ridge National Laboratory . Materials for Separation Technologies: Energy and Emission Reduction Opportunities. https://www.osti.gov/biblio/1218755 (17 March 2022, date last accessed).

[2] Humphrey JL , Keller II GE. Separation Process Technology. New York: McGraw-Hill, 1997.

[3] Sholl DS , Lively RP. Seven chemical separations to change the world. Nature 2016; 532: 435–7. 10.1038/532435a 27121824

[4] Keller GE , Marcinkowsky AE, Verma SK et al. Olefin recovery and purification via silver complexation. In: Li NN, Calo JM (eds). Separation and Purification Technology, 1st edn. New York: Marcel Dekker, Inc., 1992, 59–84.

[5] Borodziński A , Bond GC. Selective hydrogenation of ethyne in ethene-rich streams on palladium catalysts. Part 1. Effect of changes to the catalyst during reaction. Catal Rev 2006; 48: 91–144. 10.1080/01614940500364909

[6] Haszeldine RS . Carbon capture and storage: how green can black be? Science 2009; 325: 1647–52. 10.1126/science.1172246 19779187

[7] Atmospheric CO2 Data . https://scrippsco2.ucsd.edu/data/atmospheric_co2/primary_mlo_co2_record.html (17 March 2022, date last accessed).

[8] Robert R . Global Temperature Report for 2020. http://berkeleyearth.org/global-temperature-report-for-2020/ (17 March 2022, date last accessed).

[9] da Silva EF , Svendsen HF. Computational chemistry study of reactions, equilibrium and kinetics of chemical CO_2_ absorption. Int J Greenhouse Gas Control 2007; 1: 151–7. 10.1016/S1750-5836(07)00022-9

[10] Zhang Z , Yao Z-Z, Xiang S et al. Perspective of microporous metal–organic frameworks for CO_2_ capture and separation. Energy Environ Sci 2014; 7: 2868–99. 10.1039/C4EE00143E

[11] Rochelle GT . Amine scrubbing for CO_2_ capture. Science 2009; 325: 1652–4. 10.1126/science.1176731 19779188

[12] Ebner AD , Ritter JA. State-of-the-art adsorption and membrane separation processes for carbon dioxide production from carbon dioxide emitting industries. Sep Sci Technol 2009; 44: 1273–421. 10.1080/01496390902733314

[13] Dantas TLP , Amorim SM, Luna FMT et al. Adsorption of carbon dioxide onto activated carbon and nitrogen-enriched activated carbon: surface changes, equilibrium, and modeling of fixed-bed adsorption. Sep Sci Technol 2009; 45: 73–84. 10.1080/01496390903401762

[14] Takamura Y , Narita S, Aoki J et al. Evaluation of dual-bed pressure swing adsorption for CO_2_ recovery from boiler exhaust gas. Sep Purif Technol 2001; 24: 519–28. 10.1016/S1383-5866(01)00151-4

[15] Chaffee AL , Knowles GP, Liang Z et al. CO_2_ capture by adsorption: materials and process development. Int J Greenhouse Gas Control 2007; 1: 11–8. 10.1016/S1750-5836(07)00031-X

[16] Zhang J , Xiao P, Li G et al. Effect of flue gas impurities on CO_2_ capture performance from flue gas at coal-fired power stations by vacuum swing adsorption. Energy Procedia 2009; 1: 1115–22. 10.1016/j.egypro.2009.01.147

[17] Tlili N , Grévillot G, Vallières C. Carbon dioxide capture and recovery by means of TSA and/or VSA. Int J Greenhouse Gas Control 2009; 3: 519–27. 10.1016/j.ijggc.2009.04.005

[18] Park HB , Jung CH, Lee YM et al. Polymers with cavities tuned for fast selective transport of small molecules and ions. Science 2007; 318: 254–8. 10.1126/science.1146744 17932294

[19] Cannella WJ . Xylenes and ethylbenzene. In: Howe-Grant M (ed.). Kirk-Othmer Encyclopedia of Chemical Technology, 4th edn. New York: John Wiley & Sons, 1993, 831–63

[20] Wu Y , Weckhuysen BM. Separation and purification of hydrocarbons with porous materials. Angew Chem Int Ed 2021; 60: 18930–49. 10.1002/anie.202104318 PMC8453698 33784433

[21] Minceva M , Rodrigues AE. Modeling and simulation of a simulated moving bed for the separation of *p*-xylene. Ind Eng Chem Res 2002; 41: 3454–61. 10.1021/ie010095t

[22] Li Y , Yu J. Emerging applications of zeolites in catalysis, separation and host–guest assembly. Nat Rev Mater 2021; 6: 1156–74 10.1038/s41578-021-00347-3.

[23] Stiopkin IV , Weeraman C, Pieniazek PA et al. Hydrogen bonding at the water surface revealed by isotopic dilution spectroscopy. Nature 2011; 474: 192–5. 10.1038/nature10173 21654801

[24] Muller EEL , Pinel N, Laczny CC et al. Community-integrated omics links dominance of a microbial generalist to fine-tuned resource usage. Nat Commun 2014; 5: 5603. 10.1038/ncomms6603 25424998 PMC4263124

[25] Zaccai G . How soft is a protein? A protein dynamics force constant measured by neutron scattering. Science 2000; 288: 1604–7. 10.1126/science.288.5471.1604 10834833

[26] Zhang G , Keshari KR. Deuterium metabolic imaging of pancreatic cancer. NMR Biomed 2021; 34: e4603. 10.1002/nbm.4603 34369021 PMC9527824

[27] Glugla M , Lässer R, Dörr L et al. The inner deuterium/tritium fuel cycle of ITER. Fusion Eng Des 2003; 69: 39–43. 10.1016/S0920-3796(03)00231-X

[28] Iwai Y , Yamanishi T, O’hira S et al. HDT cryogenic distillation experiments at TPL/JAERI in support of ITER. Fusion Eng Des 2002; 61: 553–60. 10.1016/S0920-3796(02)00175-8

[29] Rae HK . Separation of Hydrogen Isotopes. Washington, DC: American Chemical Society, 1978.

[30] Ducret D , Ballanger A, Steimetz J et al. Hydrogen isotopes separation by thermal cycling absorption process. Fusion Eng Des 2001; 58: 417–21. 10.1016/S0920-3796(01)00475-6

[31] Beenakker JJM , Borman VD, Krylov SY. Molecular transport in subnanometer pores: zero-point energy, reduced dimensionality and quantum sieving. Chem Phys Lett 1995; 232: 379–82. 10.1016/0009-2614(94)01372-3

[32] Kim JY , Oh H, Moon HR. Hydrogen isotope separation in confined nanospaces: carbons, zeolites, metal–organic frameworks, and covalent organic frameworks. Adv Mater 2019; 31: 1805293. 10.1002/adma.201805293 30589123

[33] Crönstedt AF . Natural zeolite and minerals. Kongl Svenska Vetensk Acad handl 1756; 17: 120.

[34] Čejka J , Corma A, Zones S. Zeolites and Catalysis: Synthesis, Reactions and Applications. Weinheim: Wiley-VCH Verlag GmbH & Co. KGaA, 2010.

[35] Kulprathipanja S . Zeolites in Industrial Separation and Catalysis. Weinheim: Wiley-VCH Verlag GmbH & Co. KGaA, 2010.

[36] Xu R , Pang W, Yu J *et al.* Chemistry of Zeolites and Related Porous Materials: Synthesis and Structure. Singapore: John Wiley & Sons (Asia) Pte Ltd, 2007.

[37] Baerlocher C , McCusker LB. Database of Zeolite Structures. http://www.iza-structure.org/databases/ (17 March 2022, date last accessed).

[38] Liebau F . Structural Chemistry of Silicates: Structure, Bonding and Classification. Berlin: Springer-Verlag, 1985.

[39] Wragg DS , Morris RE, Burton AW. Pure silica zeolite-type frameworks: a structural analysis. Chem Mater 2008; 20: 1561–70. 10.1021/cm071824j

[40] Yang RT . Adsorbents: Fundamentals and Applications. New Jersey: John Wiley & Sons, 2003.

[41] Shang J , Li G, Singh R et al. Discriminative separation of gases by a ‘molecular trapdoor’ mechanism in chabazite zeolites. J Am Chem Soc 2012; 134: 19246–53. 10.1021/ja309274y 23110556

[42] Breck DW . Zeolite Molecular Sieves: Structure, Chemistry and Use. New York: John Wiley & Sons, 1974.

[43] Kuznicki SM , Bell VA, Nair S et al. A titanosilicate molecular sieve with adjustable pores for size-selective adsorption of molecules. Nature 2001; 412: 720–4. 10.1038/35089052 11507636

[44] Palomino M , Corma A, Rey F et al. New insights on CO_2_−methane separation using LTA zeolites with different Si/Al ratios and a first comparison with MOFs. Langmuir 2010; 26: 1910–7. 10.1021/la9026656 19757816

[45] Hibbe F , Caro J, Chmelik C et al. Monitoring molecular mass transfer in cation-free nanoporous host crystals of type AlPO-LTA. J Am Chem Soc 2012; 134: 7725–32. 10.1021/ja211492b 22506830

[46] Miyamoto M , Fujioka Y, Yogo K. Pure silica CHA type zeolite for CO_2_ separation using pressure swing adsorption at high pressure. J Mater Chem 2012; 22: 20186–9. 10.1039/c2jm34597h

[47] Milton RM . US Patent 1959; 2882243

[48] Reed TB , Breck DW. Crystalline zeolites. II. crystal structure of synthetic zeolite, type A. J Am Chem Soc 1956; 78: 5972–7. 10.1021/ja01604a002

[49] Liu Q , Mace A, Bacsik Z et al. NaKA sorbents with high CO_2_-over-N_2_ selectivity and high capacity to adsorb CO_2_. Chem Commun 2010; 46: 4502–4. 10.1039/c000900h 20428579

[50] Liu Y , Wu Y, Liang W et al. Bimetallic ions regulate pore size and chemistry of zeolites for selective adsorption of ethylene from ethane. Chem Eng Sci 2020; 220: 115636. 10.1016/j.ces.2020.115636

[51] Zhou Y , Zhang J, Wang L et al. Self-assembled iron-containing mordenite monolith for carbon dioxide sieving. Science 2021; 373: 315–20. 10.1126/science.aax5776 34437149

[52] Lee HS , Kim NS, Kwon D-i et al. Post-synthesis functionalization enables fine-tuning the molecular-sieving properties of zeolites for light olefin/paraffin separations. Adv Mater 2021; 33: 2105398. 10.1002/adma.202105398 34545976

[53] Jones CW , Tsuji K, Davis ME. Organic-functionalized molecular sieves as shape-selective catalysts. Nature 1998; 393: 52–4. 10.1038/29959

[54] Yan W , Hagaman EW, Dai S. Functionalization of aluminophosphate alpo4-H1 (VPI-5) with phenylphosphonic acid. Chem Mater 2004; 16: 5182–6. 10.1021/cm048766b

[55] Wu Y , Yuan D, He D et al. Decorated traditional zeolites with subunits of metal–organic frameworks for CH_4_/N_2_ separation. Angew Chem Int Ed 2019; 58: 10241–4. 10.1002/anie.201905014 31111582

[56] Wu Y , Zeng S, Yuan D et al. Enhanced propene/propane separation by directional decoration of the 12-membered rings of mordenite with ZIF fragments. Angew Chem Int Ed 2020; 59: 6765–8. 10.1002/anie.202000029 32053274

[57] Georgieva VM , Bruce EL, Verbraeken MC et al. Triggered gate opening and breathing effects during selective CO_2_ adsorption by merlinoite zeolite. J Am Chem Soc 2019; 141: 12744–59. 10.1021/jacs.9b05539 31373800

[58] Corma A , Rey F, Rius J et al. Supramolecular self-assembled molecules as organic directing agent for synthesis of zeolites. Nature 2004; 431: 287–90. 10.1038/nature02909 15372027

[59] Schreyeck L , Stumbe J, Caullet P et al. The diaza-polyoxa-macrocycle ‘Kryptofix222’ as a new template for the synthesis of LTA-type ALPO_4_: co-templating role of F^−^ and/or (CH_3_)_4_N^+^ ions. Microporous Mesoporous Mater 1998; 22: 87–106. 10.1016/S1387-1811(98)00082-1

[60] Hedin N , DeMartin GJ, Roth WJ et al. PFG NMR self-diffusion of small hydrocarbons in high silica DDR, CHA and LTA structures. Microporous Mesoporous Mater 2008; 109: 327–34. 10.1016/j.micromeso.2007.05.007

[61] Pirngruber GD , Laroche C, Maricar-Pichon M et al. Core–shell zeolite composite with enhanced selectivity for the separation of branched paraffin isomers. Microporous Mesoporous Mater 2013; 169: 212–7. 10.1016/j.micromeso.2012.11.016

[62] Miyamoto M , Ono S, Oumi Y et al. Nanoporous ZSM-5 crystals coated with silicalite-1 for enhanced *p*-xylene separation. ACS Appl Nano Mater 2019; 2: 2642–50. 10.1021/acsanm.9b00037

[63] Chao CC . US Patents 1989; 4859217

[64] Chai Y , Han X, Li W et al. Control of zeolite pore interior for chemoselective alkyne/olefin separations. Science 2020; 368: 1002–6. 10.1126/science.aay8447 32467390

[65] Liu S , Han X, Chai Y et al. Efficient separation of acetylene and carbon dioxide in a decorated zeolite. Angew Chem Int Ed 2021; 60: 6526–32. 10.1002/anie.202014680 33368904

[66] Sun M , Gu Q, Hanif A et al. Transition metal cation-exchanged SSZ-13 zeolites for CO_2_ capture and separation from N_2_. Chem Eng J 2019; 370:1450–8. 10.1016/j.cej.2019.03.234

[67] Yu Y , Li X, Krishna R et al. Enhancing CO_2_ adsorption and separation properties of aluminophosphate zeolites by isomorphous heteroatom substitutions. ACS Appl Mater Interfaces 2018; 10: 43570–7. 10.1021/acsami.8b11235 30512947

[68] Datta SJ , Khumnoon C, Lee ZH et al. CO_2_ capture from humid flue gases and humid atmosphere using a microporous coppersilicate. Science 2015; 350: 302–6. 10.1126/science.aab1680 26472904

[69] Cnudde P , Redekop EA, Dai W et al. Experimental and theoretical evidence for the promotional effect of acid sites on the diffusion of alkenes through small-pore zeolites. Angew Chem Int Ed 2021; 60: 10016–22. 10.1002/anie.202017025 PMC8251642 33496374

[70] Gi Min J , Christian Kemp K, Kencana KS et al. Dealuminated Cs-Zk-5 zeolite for propylene/propane separation. Chem Eng J 2021; 413: 127422. 10.1016/j.cej.2020.127422

[71] Barrett PA , Boix T, Puche M et al. ITQ-12: a new microporous silica polymorph potentially useful for light hydrocarbon separations. Chem Commun 2003; 39: 2114–5. 10.1039/b306440a 13678158

[72] Xiong R , Zhang L, Li P et al. Highly effective hydrogen isotope separation through dihydrogen bond on Cu(I)-exchanged zeolites well above liquid nitrogen temperature. Chem Eng J 2020; 391: 123485. 10.1016/j.cej.2019.123485

[73] Bereciartua PJ , Cantín Á, Corma A et al. Control of zeolite framework flexibility and pore topology for separation of ethane and ethylene. Science 2017; 358: 1068–71. 10.1126/science.aao0092 29170235

[74] Sala A , Pérez-Botella E, Jordá JL et al. ITQ-69: a germanium-containing zeolite and its synthesis, structure determination, and adsorption properties. Angew Chem Int Ed 2021; 60: 11745–50. 10.1002/anie.202100822 33621374

[75] Yang J , Tang X, Liu J et al. Down-sizing the crystal size of ZK-5 zeolite for its enhanced CH_4_ adsorption and CH_4_/N_2_ separation performances. Chem Eng J 2021; 406: 126599. 10.1016/j.cej.2020.126599

[76] Ackley MW , Yang RT. Adsorption characteristics of high-exchange clinoptilolites. Ind Eng Chem Res 1991; 30: 2523–30. 10.1021/ie00060a004

[77] Ackley MW , Yang RT. Diffusion in ion-exchanged clinoptilolites. AIChE J 1991; 37: 1645–56. 10.1002/aic.690371107

[78] Wang Q , Yu Y, Li Y et al. Methane separation and capture from nitrogen rich gases by selective adsorption in microporous materials: a review. Sep Purif Technol 2022; 283: 120206. 10.1016/j.seppur.2021.120206

[79] Jayaraman A , Hernandez-Maldonado AJ, Yang RT et al. Clinoptilolites for nitrogen/methane separation. Chem Eng Sci 2004; 59: 2407–17. 10.1016/j.ces.2003.10.030

[80] Jayaraman A , Yang RT, Chinn D et al. Tailored clinoptilolites for nitrogen/methane separation. Ind Eng Chem Res 2005; 44: 5184–92. 10.1021/ie0492855

[81] Du T , Fang X, Liu L et al. An optimal trapdoor zeolite for exclusive admission of CO_2_ at industrial carbon capture operating temperatures. Chem Commun 2018; 54: 3134–7. 10.1039/C8CC00634B 29527607

[82] Wang X , Yan N, Xie M et al. The inorganic cation-tailored ‘trapdoor’ effect of silicoaluminophosphate zeolite for highly selective CO_2_ separation. Chem Sci 2021; 12: 8803–10. 10.1039/D1SC00619C 34257880 PMC8246083

[83] Lozinska MM , Mangano E, Mowat JPS et al. Understanding carbon dioxide adsorption on univalent cation forms of the flexible zeolite RHO at conditions relevant to carbon capture from flue gases. J Am Chem Soc 2012; 134: 17628–42. 10.1021/ja3070864 23013547

[84] Physick AJW , Wales DJ, Owens SHR et al. Novel low energy hydrogen–deuterium isotope breakthrough separation using a trapdoor zeolite. Chem Eng J 2016; 288: 161–8. 10.1016/j.cej.2015.11.040

[85] Zhao J , Mousavi SH, Xiao G et al. Nitrogen rejection from methane via a ‘trapdoor’ K-ZSM-25 zeolite. J Am Chem Soc 2021; 143: 15195–x204. 10.1021/jacs.1c06230 34516739

[86] Thakkar H , Eastman S, Hajari A et al. 3D-printed zeolite monoliths for CO_2_ removal from enclosed environments. ACS Appl Mater Interfaces 2016; 8: 27753–61. 10.1021/acsami.6b09647 27658639

[87] Wang S , Bai P, Wei Y et al. Three-dimensional-printed core–shell structured MFI-type zeolite monoliths for volatile organic compound capture under humid conditions. ACS Appl Mater Interfaces 2019; 11: 38955–63. 10.1021/acsami.9b13819 31545028

[88] Wang S , Bai P, Sun M et al. Fabricating mechanically robust binder-free structured zeolites by 3D printing coupled with zeolite soldering: a superior configuration for CO_2_ capture. Adv Sci 2019; 6: 1901317. 10.1002/advs.201901317 PMC6724348 31508293

